# Polymer-Embedded Deep Eutectic Solvents: Mechanistic Insights into Storage and Supersaturation Stabilization

**DOI:** 10.3390/polym18060766

**Published:** 2026-03-21

**Authors:** Afroditi Kapourani, Theodora Karyofylli-Tamisoglou, Ioannis Pantazos, Maria-Emmanouela Anagnostaki, Ioannis Gkougkourelas, Panagiotis Barmpalexis

**Affiliations:** 1 Laboratory of Pharmaceutical Technology, Division of Pharmaceutical Technology, School of Pharmacy, Faculty of Health Sciences, Aristotle University of Thessaloniki, 54124 Thessaloniki, Greecepbarmp@pharm.auth.gr (P.B.); 2 Natural Products Research Centre of Excellence-AUTH (NatPro-AUTH), Center for Interdisciplinary Research and Innovation (CIRI-AUTH), 57001 Thessaloniki, Greece

**Keywords:** deep eutectic solvents, polymeric precipitation inhibitors, griseofulvin, sustained supersaturation, low-frequency NMR

## Abstract

Poor aqueous solubility remains a major limitation for the oral delivery of many active pharmaceutical ingredients (APIs). Deep eutectic solvents (DESs) exhibit remarkable drug-solubilization capacity, yet rapid precipitation upon aqueous dilution can compromise their ability to sustain supersaturation. This study investigates polymer-embedded DES (PEDES) systems as liquid supersaturating drug delivery platforms in which hydration and polymer chemistry jointly govern thermodynamic solubilization and kinetic stabilization. A choline chloride/DL-malic acid DES was prepared with 5% or 15% (*w*/*w*) water and combined with polyvinylpyrrolidone (PVP) or polyacrylic acid (PAA). Griseofulvin (GRF) was used as a precipitation-prone model drug. Structural characterization (ATR-FTIR, ^1^H-NMR), equilibrium solubility measurements, storage stability studies, and non-sink dissolution testing were conducted to elucidate formulation behavior. The DES systems enhanced GRF solubility by up to ~59-fold relative to phosphate buffer (PBS, pH 6.8). Polymer incorporation produced hydration- and concentration-dependent effects. These results suggest the presence of competitive or cooperative interaction regimes. At 5% water, PEDES formulations failed to prevent recrystallization and showed limited supersaturation maintenance. In contrast, PEDES systems containing 15% water exhibited improved stability, with the formulation containing 4% PAA sustaining elevated drug concentrations for 120 min under non-sink conditions. Low-frequency solution-state ^1^H-NMR confirmed stronger GRF–PAA interactions relative to PVP, supporting the role of polymer–drug association in supersaturation stabilization. These findings demonstrate that PEDES performance emerges from a hydration-dependent balance between solvent structuring and drug–polymer interactions, highlighting hydration and polymer functionality as key parameters for the rational design of liquid supersaturating systems.

## 1. Introduction

Poor aqueous solubility remains a major limitation in oral drug delivery and continues to hinder the successful translation of drug candidates from discovery to clinical application [1,2]. Hence, considerable effort has been devoted over recent decades to the development of formulation strategies aimed at overcoming solubility-related limitations and unlocking the therapeutic and commercial potential of poorly water-soluble drugs (PWSDs). Several formulation strategies have been proposed to address the poor aqueous solubility of drugs. These include salt formation, inclusion complexation with cyclodextrins [3,4], alternative solid-state forms (e.g., cocrystals [5]), particle size reduction [6], amorphous solid dispersions (ASDs) [7,8], and lipid-based formulations [9]. Among these approaches, deep eutectic solvents (DESs) have emerged as a promising class of liquid systems characterized by exceptionally high solubilization capacity for a broad range of organic compounds [10,11].

DESs are generally described as a subclass of eutectic systems in which the melting point depression is substantially greater than that observed for typical eutectic mixtures. DESs are generally formed by combining a hydrogen bond acceptor (HBA) and a hydrogen bond donor (HBD) in appropriate molar ratios, where strong intermolecular interactions—most commonly hydrogen bonding—between the components disrupt the crystalline lattices of the pure substances. This interaction-driven mixing results in a pronounced decrease in melting temperature, yielding a stable liquid phase at or near ambient conditions. The formation of DESs typically requires thorough mixing and mild heating to facilitate the establishment of the intermolecular hydrogen-bonding network between the HBA and HBD components [12,13,14,15,16]. DESs have been proposed as alternatives to ionic liquids (ILs). They are often considered an emerging class of green solvents, owing to their favorable attributes such as biodegradability, relatively low manufacturing cost, low volatility, and non-flammability [17,18,19]. Beyond their favorable physicochemical and environmental profiles, DESs have demonstrated remarkable drug-solubilizing capabilities [20,21]. Numerous studies have reported significant enhancement in the apparent solubility of PWSDs, in some cases reaching increases of multiple orders of magnitude [17,22,23]. This pronounced solubility enhancement has positioned DES-based drug formulations as attractive platforms for inducing drug supersaturation upon dilution in gastrointestinal (GI) fluids when administered orally.

Supersaturation provides a strong thermodynamic driving force for enhanced absorption. However, it also promotes rapid nucleation and crystal growth, increasing the risk of drug precipitation and loss of bioavailability [24]. Thus, the mechanism underlying the solubilization advantage of DESs also constitutes a critical limitation of these systems when employed without precipitation control.

To address this challenge, polymeric precipitation inhibitors (PPIs) have been explored as a means of kinetically stabilizing supersaturated drug solutions. In this vein, an advanced formulation strategy has been proposed in which the PPI is embedded directly within the DES matrix, giving rise to new polymer-embedded deep eutectic solvent (PEDES) systems [25]. This approach redefines conventional supersaturating drug delivery systems by integrating polymer-based precipitation control directly within the solubilizing matrix.

Unlike ILs, which rely on fully ionic architectures and often exhibit distinct physicochemical and toxicological considerations [26], DESs are constructed from molecular hydrogen-bonding networks formed between neutral or weakly ionic components. Consequently, polymer incorporation within PEDES systems does not generate a new ionic phase. Instead, it reshapes an existing, highly cooperative hydrogen-bonded solvent structure. Furthermore, PEDES systems differ fundamentally from ASDs, where the polymer primarily stabilizes a high-energy amorphous solid phase by suppressing recrystallization within a glassy matrix [27,28]. In contrast, PEDES formulations operate entirely in the liquid state, where drug stabilization arises from dynamic solvent structuring and reversible intermolecular association rather than vitrification. Similarly, PEDES systems are mechanistically distinct from simple cosolvent-based supersaturating systems. In cosolvent approaches, the drug is solubilized by polarity modulation, and precipitation inhibition occurs predominantly after dilution through polymer–drug interactions in the aqueous phase [29]. In PEDES systems, polymer incorporation modifies the intrinsic solvent architecture prior to dilution. As a result, the hierarchy of intermolecular interactions within the eutectic matrix is altered. Supersaturation generation and stabilization therefore emerge from a pre-organized multicomponent liquid network rather than from post-dilution kinetic intervention alone. Hence, PEDESs can be considered a mechanistically unique class of polymer-based supersaturated drug delivery systems, in which hydration-controlled solvent organization and polymer-mediated interaction redistribution jointly govern thermodynamic solubilization and kinetic stabilization.

In this context, the present study moves beyond solubility enhancement and seeks to mechanistically evaluate the structural and kinetic behavior of drug-loaded PEDES formulations. Specifically, we systematically examine how controlled hydration and polymer chemistry modulate thermodynamic solubilization, molecular organization, and supersaturation performance in PEDES-based drug delivery systems. By deliberately challenging the PEDES platform with griseofulvin (GRF)—a model active pharmaceutical ingredient (API) characterized by pronounced instability in the supersaturated state, leading to rapid precipitation [30]—we aim to elucidate the interaction regimes that determine whether polymer incorporation disrupts, redistributes, or cooperatively enhances drug stabilization. Through this approach, the work aims to establish a design principle for similar polymer-embedded DES supersaturated drug delivery systems.

## 2. Materials and Methods

### 2.1. Materials

GRF (C_17_H_19_ClO_6_, molecular weight 354.78 g/mol, purity ≥ 97.0%) was purchased from Thermo Fisher Scientific (Waltham, MA, USA). Choline Chloride (ChCl, purity ≥ 99%), used as the HBA, and DL-Malic acid (DL-Mal) (purity ≥ 99+%), used as the HBD for the preparation of the DES formulations, were obtained from Sigma-Aldrich (Steinheim, Germany). PVP, Kollidon^®^ K12 (PVP K12), with an average molecular weight of approximately 3000 g/mol, was supplied by BASF (Ludwigshafen, Germany). PAA (molecular weight 1800 g/mol) was purchased from Sigma-Aldrich (St. Louis, MO, USA). All other chemicals and reagents were of analytical or pharmaceutical grade and were used as received.

### 2.2. Preparation of DESs

DESs were prepared using a conventional heating and stirring method [31,32]. Specifically, ChCl was mixed with DL-Mal at a defined molar ratio of 1:1. Prior to preparation, all DES components were dried for 24 h to eliminate residual moisture. Accurately weighed quantities of the dried components were then transferred into capped glass bottles and heated on a hot plate at 80 °C under continuous stirring for 2 h, until a clear and homogeneous liquid was obtained.

After heating, the resulting liquids were cooled to room temperature (25 °C). Subsequently, water was added to the prepared DES formulations at concentrations of 5% *w*/*w* and 15% *w*/*w*, respectively, to modulate viscosity and evaluate the influence of water content on formulation performance. The mixtures were stirred until complete homogenization was achieved.

### 2.3. Polymer Solubility in DES Formulations

The solubility of the selected PPIs in the ChCl/DL-Mal DESs was assessed by incremental polymer addition. Τhe weight percentage of polymer added relative to the total weight of the DES system (containing either 5% *w*/*w* or 15% *w*/*w* water) was initially set at 0.5% *w*/*w* and subsequently increased in increments of 0.5% *w*/*w*. After each addition, the formulations were equilibrated and visually inspected. Visual assessment was selected as the primary criterion, as turbidity onset in DES systems is strongly correlated with macroscopic phase separation and loss of homogeneity. Polymer solubility was defined as the highest polymer concentration at which a clear and homogeneous system was maintained, whereas the appearance of turbidity was considered indicative of polymer precipitation or phase separation.

### 2.4. Structural Properties of Prepared DESs and PEDES

The structural properties of the prepared DESs, PEDESs and their components were examined through attenuated total reflectance Fourier transform infrared (ATR-FTIR) and proton nuclear magnetic resonance (^1^H-NMR) spectroscopy.

#### 2.4.1. ATR-FTIR Measurements

ATR-FTIR measurements of the samples were carried out using a Shimadzu IR-Prestige-21-FT-IR infrared spectrometer (Tokyo, Japan) coupled with a horizontal Golden Gate MKII single-reflection ATR system (Specac, Kent, UK) equipped with ZnSe lenses. Each spectrum was recorded following appropriate background subtraction. The average spectrum of sixty-four (64) successive scans in the region of 700–4000 cm^−1^ (at a resolution of 4 cm^−1^) was recorded. Spectral acquisition and data processing were performed using IRsolution software (version 1.30, Shimadzu, Tokyo, Japan).

#### 2.4.2. ^1^H-NMR Spectroscopy

^1^H-NMR spectra were acquired using a low-frequency (60 MHz) benchtop NMR spectrometer (Spinsolve, Magritek, Germany) and processed with Spinsolve software v2.6.10. Spectral assignments of the raw materials (GRF and individual PEDES components) were established based on standard two-dimensional NMR experiments, including correlation spectroscopy, and were cross-validated against published reference data. Prior to data acquisition, the spectrometer was shimmed using a reference sample consisting of a mixture of 3% (*v*/*v*) deionized H_2_O in D_2_O, contained in a standard 5 mm outer-diameter NMR tube. This procedure optimized magnetic field homogeneity, yielding peak widths of approximately 0.21 Hz at 50% peak height and 6.72 Hz at 0.55% peak height. All samples were recorded using DMSO (1:12 *w*/*w* sample to diluent ratio).

### 2.5. API Thermodynamic Solubility in DESs and PEDES

The thermodynamic solubility of the API in both DES and PEDES formulations was evaluated by introducing an excess amount of GRF into each system. The mixtures were agitated using a magnetic stirrer at 350 rpm and maintained at 25 °C for a period of 24 h to allow equilibration to be achieved. Following equilibration, aliquots were transferred into 2 mL microcentrifuge tubes and subjected to centrifugation at 10,000 rpm for 30 min. This procedure was performed twice to ensure effective removal of undissolved material.

The resulting supernatants were carefully collected and filtered through 0.45 μm Polyvinylidene Fluoride (PVDF) syringe filters to eliminate residual particulates. Prior to analysis, the filtered samples were diluted 1:1000 (*v*/*v*) with methanol and subsequently quantified using high-performance liquid chromatography (HPLC), as described in the relevant analytical section. All experiments were conducted in triplicate (*n* = 3).

### 2.6. Preparation of Drug-Loaded PEDES

GRF-loaded PEDES formulations were prepared by incorporating GRF at a concentration corresponding to 100% of its experimentally determined equilibrium solubility in the selected DES systems containing pre-dissolved PPIs (PVP or PAA) at the previously optimized concentrations. The formulations were stirred under controlled conditions until optically clear and homogeneous solutions were obtained, confirming complete drug solubilization.

After equilibration, any residual undissolved GRF was removed by centrifugation at 10,000 rpm for two 30 min cycles. The resulting drug-loaded PEDES formulations were transferred to sealed glass vials and stored over phosphorus pentoxide (P_2_O_5_) in desiccators to protect against moisture uptake until further characterization.

### 2.7. Non-Sink Drug Miniature-Scale Release

Miniature-scale non-sink drug release experiments were conducted to evaluate the supersaturation and precipitation behavior of DES- and PEDES-based formulations based on previously published methodology [25]. These non-sink experiments were designed as comparative supersaturation stress tests rather than biopredictive dissolution studies. In brief, samples consisting of 1 mL of DES, either polymer-free or containing the selected polymeric precipitation inhibitor, were loaded with the API at 100% of its experimentally determined equilibrium solubility. This drug loading level was selected as a reference supersaturation condition commonly employed for comparative assessment of non-sink release performance across formulations. The samples were initially stirred at 37 °C for 24 h in hermetically sealed 20 mL glass vials to ensure equilibration.

Following equilibration, the vials were transferred to an oven maintained at 37 °C. At time zero, 10 mL of pH 6.8 phosphate buffer, pre-heated to 37 °C, was added to each vial to initiate drug release. To maintain isothermal conditions throughout the experiment, all buffers, syringes, and filters were pre-heated prior to use.

At predetermined time points (2, 5, 10, 15, 30, 60, 90, and 120 min), aliquots were withdrawn using a 1 mL syringe fitted with a needle, which was used to pierce the rubber septum of the vial. An equal volume of fresh, pre-warmed dissolution medium was added back into the vessel. Each sample withdrawn was immediately centrifuged at 10,000 rpm for 10 min and subsequently diluted with methanol prior to analysis. The concentration of API in the release medium at each time point was quantified using HPLC, and the resulting concentration–time profiles were plotted accordingly. All release experiments were performed in triplicate for each formulation.

### 2.8. Quantification of GRF via HPLC

GRF concentrations were quantified using HPLC (Agilent 1260 Infinity II, Agilent Technologies, Santa Clara, CA, USA) employing a published analytical method [33]. Chromatographic separation was achieved on a reversed-phase C18 column (Waters Symmetry^®^, 250 mm × 4.6 mm i.d., 5 μm particle size) under isocratic elution conditions using a mobile phase consisting of methanol and water (70:30, *v*/*v*). The flow rate was set at 1.0 mL/min, with an injection volume of 20 μL and a column temperature maintained at 30 °C. GRF detection was carried out at a wavelength of 291 nm. Quantification was based on an external calibration curve constructed using standard GRF solutions over a concentration range of 0.01–100 ppm.

### 2.9. Stability Testing

The physical stability of the DES-based formulations, both polymer-free and polymer-containing, was investigated over a four-week storage period under controlled environmental conditions. Samples were stored in open glass containers at ambient temperature (25 °C) and exposed to a high relative humidity (RH) environment (60%).

During the storage period, the formulations were regularly monitored for indications of physical instability, such as phase separation, crystallization, precipitation, or color changes. Polarized light microscopy (PLM; Olympus BX41, Olympus, Tokyo, Japan) was employed to support visual inspection and to enable detection of crystalline domains and subtle structural changes. At the end of the study, gravimetric measurements were performed to determine changes in sample mass, allowing quantification of moisture uptake or loss and providing insight into the hygroscopic behavior and stability of the formulations.

### 2.10. Statistical Analysis

Statistical analysis was performed in SPSS Statistics 29 (IBM, Armonk, NY, USA) using analysis of variance (ANOVA) with the General Linear Model, followed by comparisons of means conducted with Tukey’s test. Differences were considered statistically significant at *p* < 0.05. Results are expressed as mean ± SD of three independent experiments.

## 3. Results and Discussion

### 3.1. Preparation of PEDESs

The DES employed in this study consisted of a eutectic mixture of ChCl and DL-Mal at a 1:1 molar ratio, incorporating a controlled amount of water. Water was deliberately introduced to enhance molecular mobility, facilitate the solubilization of the PPIs, and improve key physicochemical properties, particularly viscosity. In addition, water incorporation improved handling characteristics and reduced preparation time without compromising the integrity of the eutectic structure, in line with previous reports [34,35].

Importantly, the water content was carefully controlled. Excessive water levels can weaken or disrupt the hydrogen-bonding network of DESs, reducing solvent strength and increasing the risk of polymer and/or drug precipitation during preparation or storage. Consequently, precise control of water content is critical to achieving an optimal balance between solubilization capacity and supersaturation stability. On this basis, DES-based systems containing two distinct water levels (5% and 15% *w*/*w*) were investigated in the present study in order to elucidate the influence of water content on the performance and precipitation behavior of the resulting PEDES formulations.

**Figure 1:**
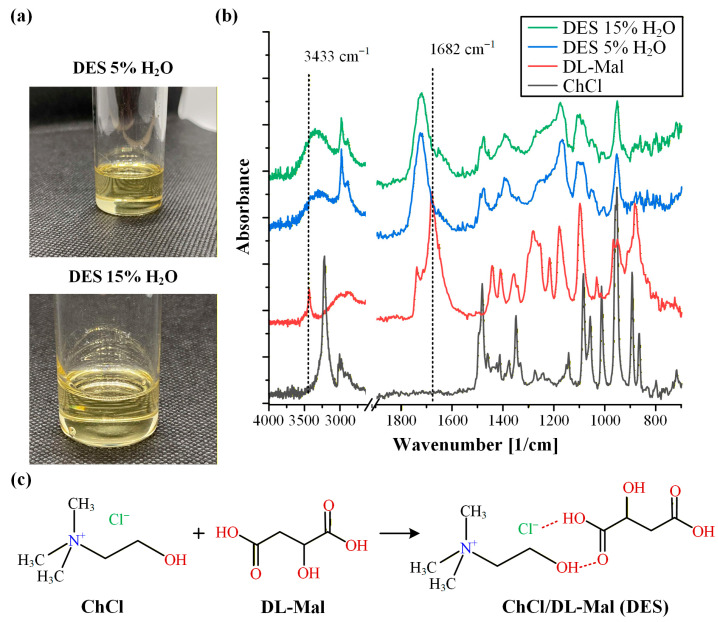
Images of the ChCl/DL-Mal DESs prepared with 5% and 15% *w*/*w* of water (**a**), ATR-FTIR spectra of the raw materials and the prepared ChCl/DL-Mal DESs (**b**), and scheme of the ChCl/DL-Mal DES preparation (**c**).

The prepared ChCl/DL-Mal DESs appeared as slightly yellow, transparent liquids with satisfactory fluidity (Figure 1a). It should be noted that the yellow coloration observed was expected, as DES systems developed using DL-Mal have been previously reported to exhibit a yellowish appearance [36,37]. In particular, ChCl/DL-Mal DES prepared at a 1:1 molar ratio has been previously studied and reported to display this characteristic color, while it was also observed that increasing the water content progressively improved the transparency of the system [38]. Accordingly, compared with previously reported ChCl/DL-Mal DESs, which have been described as colorless when prepared with higher water contents [39], the faint yellow coloration observed in the present study is likely attributable to the lower water levels employed (5% and 15% *w*/*w*, as opposed to approximately 30% *w*/*w* in earlier studies). This observation suggests that water content may influence not only the physicochemical properties but also the optical characteristics of the DES.

The formation of the DES was confirmed using ATR-FTIR spectroscopy. Figure 1b presents the ATR-FTIR spectra of ChCl, DL-Mal, and the corresponding DESs containing 5% and 15% *w*/*w* of water. The spectrum of ChCl exhibited characteristic bands corresponding to symmetric bending vibrations of -CH_3_ at 1481 cm^−1^, -CH_3_ stretching vibrations in the ~2800–3000 cm^−1^ region, and -OH stretching at 3217 cm^−1^. In parallel, DL-Mal displayed a broad -OH stretching band at 3433 cm^−1^ and a C=O stretching vibration at 1682 cm^−1^. Upon DES formation, the characteristic C=O and -OH absorption bands of DL-Mal were shifted from 1682 cm^−1^ and 3433 cm^−1^ to 1722 cm^−1^ and 3331 cm^−1^, respectively. These spectral shifts are consistent with previously reported data and provide strong evidence for the establishment of a hydrogen-bonded eutectic network between ChCl and DL-Mal [39]. Specifically, they indicate hydrogen-bond interactions involving the carboxyl group of DL-Mal and the chloride anion and/or hydroxyl functionalities of ChCl, as schematically illustrated in Figure 1c.

**Figure 2:**
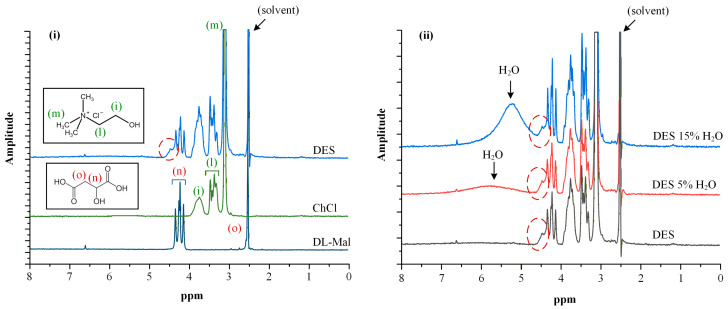
Low-frequency ^1^H-NMR spectra of the prepared DESs and the raw materials used (**i**), along with the DESs after the addition of 5% and 15% of H_2_O (**ii**).

In addition to ATR-FTIR analysis, low-frequency ^1^H-NMR spectroscopy was employed to further verify the formation of the ChCl/DL-Mal deep eutectic solvent. Figure 2(i) presents the ^1^H-NMR spectra of the individual raw materials (ChCl and DL-Mal) alongside that of the prepared DES. For ChCl, characteristic proton resonances were observed at δ = 3.74 ppm (-N^+^(CH_3_)_3_), 3.46–3.30 ppm (-CH_2_- adjacent to the hydroxyl group), and 3.10 ppm, while DL-Mal exhibited signals at δ = 4.36–4.16 ppm (-CH-) and 2.58–2.94 ppm (-CH_2_-), consistent with previously reported data [40,41].

In the spectrum of the ChCl/DL-Mal DES, the appearance of an additional shoulder at δ~4.47 ppm was observed. This spectral modification, not present in the individual components, indicates alteration of the local proton environment and is consistent with the establishment of hydrogen-bond interactions between the HBA (ChCl) and HBD (DL-Mal). The emergence of this feature provides further evidence for the formation of a new, structured eutectic phase rather than a simple physical mixture of the constituents.

Importantly, incorporation of 5% or 15% *w*/*w* water into the DES did not produce substantial shifts or new resonances in the ^1^H-NMR spectra (Figure 2(ii)), suggesting preservation of the underlying hydrogen-bonded network. The only additional signal observed was a broad peak at δ~4.50–6.50 ppm corresponding to water protons. The absence of significant spectral perturbations upon hydration indicates that, within the investigated range, water acts primarily as a plasticizing and mobility-enhancing component without disrupting the fundamental eutectic structure. Collectively, the ^1^H-NMR data corroborate the successful formation of the DES across all compositions studied.

Following the proof of DES formation, PPIs were incorporated to generate the corresponding PEDES systems. Two chemically distinct polymers (PVP and PAA) were selected to systematically investigate how differences in hydrogen-bonding functionality and interaction strength influence polymer compatibility and precipitation inhibition within the DES matrix. PVP is a neutral polymer that interacts primarily as a strong hydrogen-bond acceptor through its carbonyl moieties, whereas PAA is an acidic polymer that combines moderate hydrogen-bond acceptor capability with pronounced hydrogen-bond donor functionality arising from its carboxylic acid groups [42]. This fundamental contrast in interaction profiles was expected to differentially affect polymer–DES and polymer–drug association phenomena.

PEDES formulations were prepared by progressively incorporating each polymer into the preformed DESs, starting at 0.5% *w*/*w* and increasing stepwise in 0.5% *w*/*w* increments, followed by overnight stirring to ensure complete dissolution and equilibration. Based on this screening, polymer contents of 2% *w*/*w* and 4% *w*/*w* for both PVP and PAA were identified as optimal, as these systems remained optically transparent and free of phase separation. These concentrations were therefore selected for subsequent PEDES preparation and performance evaluation, providing a suitable window for assessing the influence of polymer chemistry on supersaturation stabilization without compromising formulation integrity.

**Figure 3:**
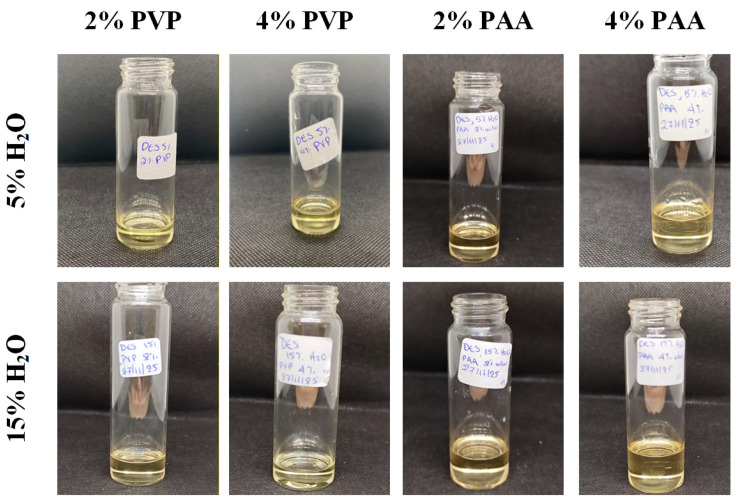
Macroscopic images of neat (without the addition of the API) PEDESs immediately after their preparation having either 5% or 15% *w*/*w* of water.

For the preparation of PEDES formulations used in the subsequent experiments, the required quantities of polymers were accurately weighed to correspond to 2% or 4% *w*/*w* of the final system and added directly to the pre-prepared DES. The mixtures were then stirred at 300 rpm at room temperature until complete dissolution of the polymer was achieved (approximately 6 h), resulting in homogeneous and optically clear PEDES systems prior to further characterization (Figure 3).

### 3.2. Physical Stability of PEDESs

The incorporation of additional components into DES systems has been reported to compromise their structural integrity and physical stability, potentially leading to phase separation or crystallization phenomena [25,43]. In this context, systematic evaluation of PEDES stability was considered essential in order to assess the impact of polymer incorporation on the long-term integrity of the eutectic matrix. Accordingly, PEDES samples (~20 mL) were prepared and stored for four weeks under controlled environmental conditions (25 °C, 60% relative humidity), selected to reflect standard pharmaceutical storage conditions in accordance with International Council for Harmonisation guidelines. Throughout the storage period, formulations were periodically inspected for macroscopic signs of physical instability. Complementary PLM analysis was employed to detect the emergence of crystalline domains and subtle microstructural alterations that may not be evident by visual inspection alone. In parallel, gravimetric analysis was conducted to quantify changes in sample mass, providing insight into moisture uptake or loss and its potential contribution to PEDES stability during storage.

**Figure 4:**
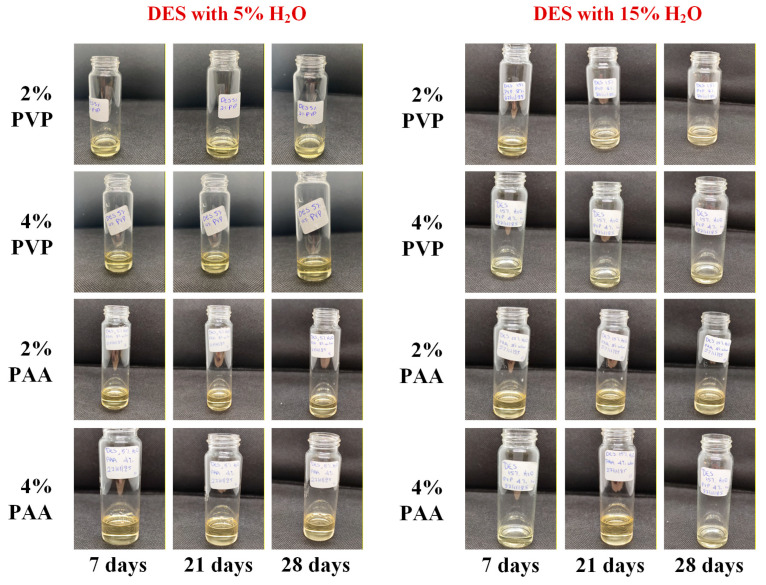
Macroscopic images of the neat PEDESs (i.e., without the addition of the API) during the storage stability study.

Figure 4 presents representative macroscopic images of the prepared PEDES formulations during the storage period, while the corresponding PLM micrographs are shown in Figure S1. Across all formulations and storage time points examined, the PEDES systems remained physically stable, exhibiting no visible signs of phase separation, precipitation, crystallization, or color change. Consistent with the macroscopic observations, PLM analysis did not reveal the presence of birefringent domains or other microstructural features indicative of crystalline phase formation. Collectively, these findings demonstrate that incorporation of the selected polymers at the investigated concentrations did not compromise the physical stability of the DES matrix under the applied storage conditions.

Gravimetric analysis of the PEDES formulations during storage at 25 °C and 60% RH is summarized in Table 1 and provides further insight into their physical stability and moisture sensitivity. Across all formulations, only minor changes in sample mass were observed, with weight variations remaining below 0.02%. Notably, all PEDES systems exhibited a slight net weight loss rather than weight gain, indicating the absence of hygroscopic moisture uptake under the applied storage conditions. This behavior suggests that the DES matrix, even after polymer incorporation, remains structurally robust and resistant to environmental moisture.

Formulations containing 15% *w*/*w* water displayed marginally higher mass losses compared with their 5% *w*/*w* counterparts, consistent with partial evaporation or redistribution of loosely bound water rather than destabilization of the eutectic network. Differences between polymer types were comparatively small, although PEDES containing PAA tended to exhibit slightly higher weight loss than PVP-based systems at equivalent water contents, which may be attributed to differences in hydrogen-bonding functionality and internal water mobility within the matrix. Importantly, increasing polymer concentration from 2% to 4% *w*/*w* did not lead to systematic increases in mass change, indicating that polymer loading within this range does not adversely affect PEDES stability. Collectively, these findings corroborate the visual and microscopic observations and confirm that the investigated PEDES formulations maintain excellent physical stability under pharmaceutically relevant storage conditions.

### 3.3. GRF Incorporation into the PEDES Formulations

Following confirmation of the physical stability of the polymer-containing DES systems, the incorporation of GRF into the PEDES formulations was investigated. Given the pronounced crystallization tendency and limited supersaturation stability of GRF, its successful incorporation represents a stringent test of the solubilization capacity and kinetic stabilization potential of the PEDES platform. As an initial step, the saturation solubility of GRF was determined in all DES- and PEDES-based systems and benchmarked against its solubility in PBS, pH 6.8, which served as a physiologically relevant aqueous reference medium (Table 2).

As anticipated, GRF exhibited very low aqueous solubility in PBS (9.01 ± 1.03 μg/mL), confirming its classification as a PWSD and validating its selection as a model drug compound for supersaturation studies. In contrast, incorporation into the ChCl/DL-Mal DES systems resulted in a dramatic enhancement of solubility. Similar solubility enhancement has been widely reported for PWSDs in DES systems. Previous studies have demonstrated that DES matrices can substantially increase API solubility by modifying the hydrogen-bonding environment and reducing the activity coefficient of the solute, in some cases resulting in increases of several orders of magnitude [22,23]. The polymer-free DES containing 5% *w*/*w* of water achieved a GRF solubility of 531.13 ± 11.40 μg/mL, corresponding to an approximately 59-fold increase relative to PBS. Increasing the water content to 15% *w*/*w* reduced GRF solubility to 417.90 ± 6.29 μg/mL, although this still represents an approximately 46-fold enhancement over aqueous buffer. The reduction in GRF solubility upon increasing water content from 5% to 15% *w*/*w* reflects a measurable attenuation of the eutectic solvent strength. At elevated hydration levels, competitive hydrogen bonding between water and DES constituents partially weakens the structured ChCl/DL-Mal interaction network, reducing the cohesive energy density of the medium. This restructuring diminishes preferential solvation of GRF within the DES microenvironment, leading to a lower thermodynamic solubility. Thus, water acts not merely as a viscosity modifier but as a regulator of solvent organization and drug stabilization capacity.

Polymer incorporation produced formulation-dependent effects on GRF solubility, indicating that PEDES systems are governed by a complex interplay between drug-DES, drug–polymer, and polymer–DES interactions. In the 5% *w*/*w* water systems, incorporation of 2% PVP reduced GRF solubility to 486.03 ± 10.83 μg/mL, whereas increasing PVP to 4% *w*/*w* restored solubility (513.19 ± 12.72 μg/mL) to values approaching that of the neat DES. At lower polymer loading, PVP introduces additional carbonyl hydrogen-bond acceptor sites that compete with GRF for association with the hydrogen-bond donor functionalities of DL-Mal, thereby redistributing intermolecular interactions within the eutectic matrix. At higher polymer concentrations, however, the increased density of carbonyl groups enables sufficient direct drug–polymer association to offset this competitive effect, restoring solubilization capacity. This concentration-dependent transition reflects a shift from interaction redistribution to cooperative stabilization within the multicomponent network.

In contrast, incorporation of PAA in the 5% water systems led to a more pronounced reduction in GRF solubility (428.61 ± 9.56 μg/mL at 2% and 445.30 ± 6.58 μg/mL at 4%). Owing to its carboxylic acid functionality, PAA introduces strong hydrogen-bond donor capacity into an already donor-rich eutectic environment. This enhances polymer–DES association, reinforcing internal network cohesion but reducing availability of favorable drug–DES interaction sites. Within this structural regime, polymer–matrix interactions dominate over drug–polymer stabilization, resulting in decreased apparent solubility.

A more complex behavior was observed in the 15% *w*/*w* water systems. At 2% polymer concentration, both PVP and PAA reduced GRF solubility (363.66 ± 8.29 and 341.77 ± 7.08 μg/mL, respectively) relative to the polymer-free DES. This suggests that under partially hydrated conditions, the DES network becomes more sensitive to additional hydrogen-bonding components. At low polymer concentrations, this effect may disrupt the solvent structure while providing insufficient stabilization of the drug. Interestingly, at 4% polymer loading, divergent trends emerged. While 4% PVP moderately increased solubility to 457.65 ± 9.31 μg/mL, 4% PAA produced a marked enhancement, reaching 501.29 ± 5.50 μg/mL, exceeding even the neat 15% DES. This observation indicates that at sufficient concentration, PAA can transition from acting as a network disruptor to functioning as an auxiliary solubilizing matrix component. Under higher hydration conditions, increased polymer concentration may promote formation of a cooperative hydrogen-bonding microenvironment that enhances GRF stabilization through combined DES-polymer interactions.

Collectively, these findings demonstrate that PEDES systems behave as adaptive multicomponent hydrogen-bonding networks in which solubilization performance emerges from a balance between solvent structuring, competitive association, and cooperative stabilization. Three interaction regimes can be distinguished: (i) a DES-dominated regime in which GRF solubility is governed primarily by optimized ChCl/DL-Mal organization; (ii) an interaction-redistribution regime at low polymer loadings, where additional hydrogen-bond sites perturb the established solvation structure; and (iii) a cooperative regime at higher polymer concentrations, where sufficient drug–polymer association compensates for network perturbation and restores or enhances solubilization. Importantly, the transition between these regimes is strongly modulated by water content, underscoring the central role of hydration in determining PEDES thermodynamic behavior.

Notably, enhanced thermodynamic solubility alone does not guarantee supersaturation stability upon dilution. In fact, systems exhibiting slightly reduced thermodynamic solubility may still demonstrate superior precipitation inhibition due to kinetic stabilization effects mediated by polymer–drug association in the aqueous phase. Therefore, these equilibrium solubility data must be interpreted in conjunction with the following non-sink release studies to fully elucidate PEDES performance.

### 3.4. Physical Stability of GRF-Loaded PEDES Formulations

Drug incorporation may alter the internal hydrogen-bonding network and microstructural organization of DES-based systems, potentially increasing the risk of phase separation, crystallization, or drug precipitation during storage. Therefore, stability assessment of GRF-loaded PEDESs was conducted under conditions analogous to those applied to the neat PEDES formulations in order to directly assess the impact of drug loading on formulation integrity and to confirm the robustness of the PEDES platform in the presence of a crystallization-prone API.

**Figure 5:**
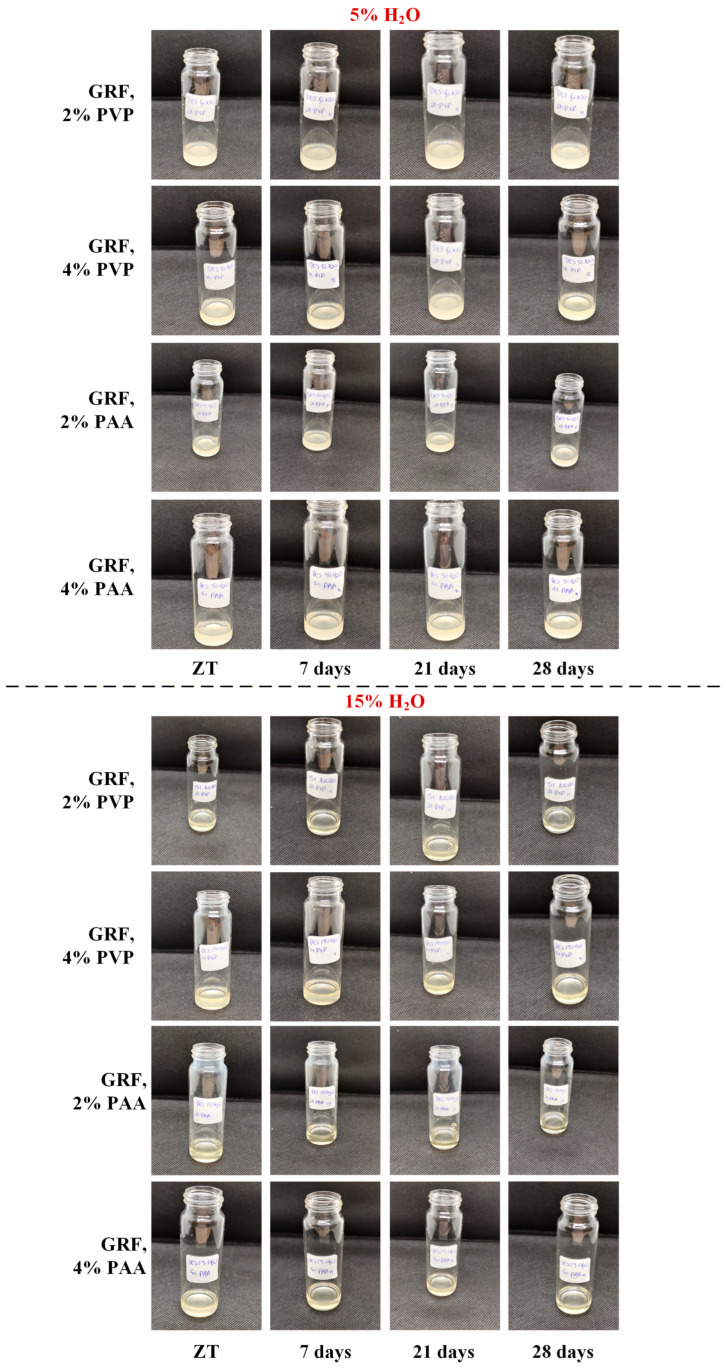
Macroscopic images of the GRF-loaded PEDESs during the storage stability study for up to 28 days.

**Figure 6:**
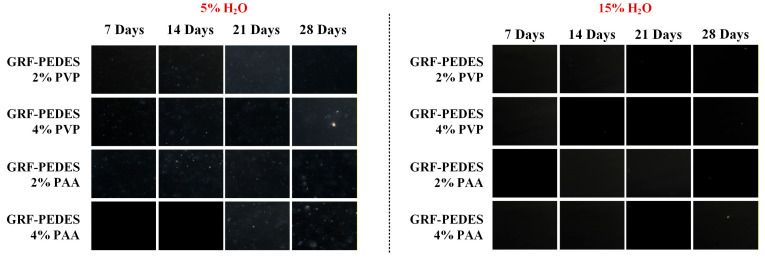
PLM micrographs of the GRF-loaded PEDESs during the storage stability study for up to 28 days.

Macroscopic (Figure 5) and microscopic (Figure 6) evaluation revealed a clear dependence of physical stability on the water content of the DES matrix. For formulations containing 5% *w*/*w* water, phase separation was observed during the storage studies from the first week of storage, indicating the presence of a solid phase in all samples. PLM analysis (Figure 6) confirmed that this solid phase corresponded to recrystallized GRF, as evidenced by the appearance of birefringent crystalline domains in the corresponding micrographs. Notably, GRF crystals were consistently observed in all 5% water formulations throughout the entire storage period of 28 days, indicating that polymer incorporation at the investigated concentrations was insufficient to kinetically stabilize the supersaturated drug state under these low-hydration conditions.

In contrast, a markedly different stability profile was observed for GRF-loaded PEDES formulations containing 15% *w*/*w* water. At zero time, all samples appeared optically clear and homogeneous, with no detectable precipitates (Figure 6). The absence of GRF recrystallization was further confirmed by PLM analysis, which revealed no birefringent features indicative of crystalline drug. Importantly, this stability was maintained throughout the entire storage period, as no signs of GRF precipitation or crystallization were detected in any of the 15% *w*/*w* water PEDES formulations at any time point examined (Figure 6). These findings indicate that a minimum hydration threshold is required to enable sufficient molecular mobility and interaction reorganization within the PEDES matrix to kinetically suppress GRF nucleation. Below this threshold, limited structural flexibility restricts dynamic drug–polymer association, favoring recrystallization.

The results of the gravimetric analysis of the GRF-loaded PEDES formulations stored for 28 days are presented in Table 3. Across all formulations, only minimal mass changes were observed, with relative weight losses remaining below 0.02%, indicating the absence of significant moisture uptake or formulation degradation following drug incorporation. Importantly, the direction and magnitude of the mass changes closely mirrored those observed for the corresponding neat PEDES systems, suggesting that GRF loading does not fundamentally alter the moisture sensitivity or environmental response of the DES-polymer matrix.

As expected, formulations containing 15% *w*/*w* water exhibited higher weight losses (≈0.011–0.012%) compared with their 5% *w*/*w* counterparts (≈0.003–0.006%). This behavior is consistent with partial evaporation or redistribution of loosely bound water rather than structural destabilization of the PEDES network. Notably, the presence of GRF did not amplify this effect, indicating that the drug does not promote additional water loss or disrupt hydrogen-bonding interactions governing water retention within the system. Differences between polymer types were minor, with both PVP- and PAA-containing formulations exhibiting comparable mass changes at equivalent water contents, further supporting the compatibility of both polymers with the GRF-loaded PEDES architecture. Within each hydration level, increasing polymer concentration from 2% to 4% *w*/*w* did not lead to systematic increases in weight loss, demonstrating that polymer loading within the investigated range does not adversely affect storage stability in the presence of the drug. These results confirm that GRF incorporation does not compromise the intrinsic stability of PEDES formulations and reinforce the critical role of controlled water content in maintaining long-term stability of drug-loaded systems.

### 3.5. Molecular Interactions via ATR-FTIR

To elucidate the molecular basis underlying the distinct physical stability profiles observed among the GRF-loaded PEDES formulations, the intermolecular interactions between the DES components, PPIs, and GRF were investigated using ATR-FTIR spectroscopy.

**Figure 7:**
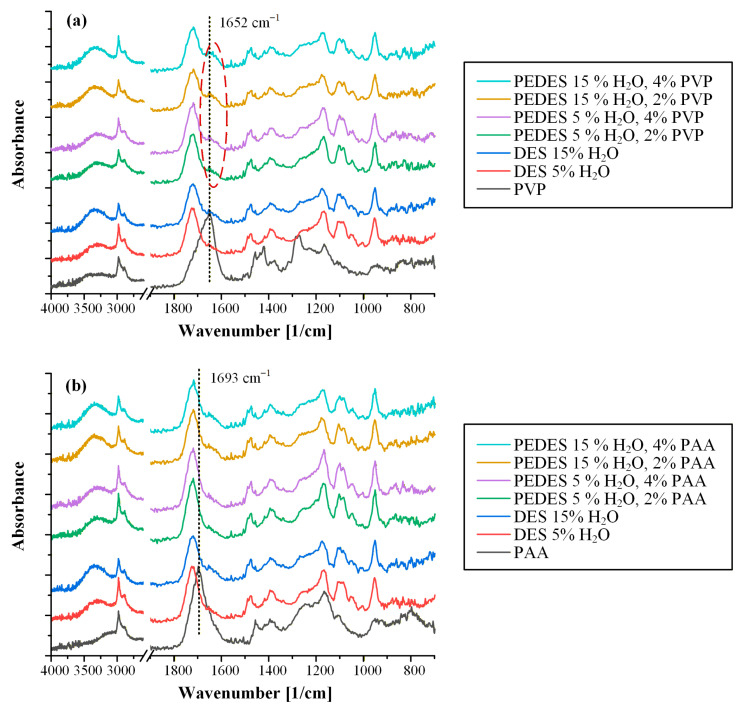
ATR-FTIR spectra of neat PEDESs containing PVP (**a**) or PAA (**b**) as PPIs.

Figure 7 presents the ATR-FTIR spectra of the neat PEDES formulations, prepared in the absence of GRF, together with those of the individual raw materials, namely PVP, PAA, and the ChCl/DL-Mal DESs containing 5% and 15% *w*/*w* of water.

The PPI spectra exhibited characteristic structural features. PVP exhibited a prominent absorption band at 1652 cm^−1^, corresponding to the C=O stretching vibration of the lactam moiety (Figure S2(i), Supplementary Material), which is well known to act as a strong hydrogen-bond acceptor. In contrast, PAA displayed a characteristic absorption band centered at 1693 cm^−1^, attributed to the C=O stretching vibration of the carboxylic acid groups (Figure S2(ii), Supplementary Material) and the formation of stable intermolecular cyclic dimers within the polymeric backbone. In addition, both polymers showed a broad absorption band in the region between 3500 and 3000 cm^−1^, which can be ascribed to OH stretching vibrations associated with the absorbed or structurally bound water in both polymers and the -COOH stretching vibration of PAA.

Examination of the ATR-FTIR spectra of the PVP-containing PEDES formulations (Figure 7a) revealed the presence of a shoulder-like peak at approximately 1652 cm^−1^ (highlighted in the spectra by a red dashed circle). This feature coincides with the characteristic C=O stretching vibration of PVP and confirms successful incorporation of the polymer within the DES matrix. The low intensity of this band is consistent with the limited polymer content (2 or 4% *w*/*w*) and its molecular dispersion within the hydrogen-bond-rich DES matrix. Importantly, the persistence of this spectral feature across both hydration levels suggests that PVP remains molecularly integrated within the PEDES network rather than undergoing phase separation or aggregation, supporting its role as an active component in precipitation inhibition.

In contrast, for PEDES formulations containing PAA as the polymeric additive (Figure 7b), the characteristic C=O stretching band of PAA at 1693 cm^−1^ could not be distinctly resolved in the composite spectra. This observation is most plausibly attributed to spectral overlap between the PAA carbonyl band and the dominant C=O stretching vibration of the DESs, observed at approximately 1722 cm^−1^. Given the close proximity of these bands and the relatively low PAA concentration in the PEDES formulations, discrimination of the polymer-specific signal becomes inherently challenging. Accordingly, the absence of a resolved PAA carbonyl peak does not indicate lack of incorporation but rather spectral overlap with the dominant DES carbonyl band.

Moving now to the drug-loaded PEDESs, and in order to facilitate the interpretation, the spectrum of crystalline GRF was first examined as a reference. Figure S3 (Supplementary Material) shows characteristic sharp, well-resolved absorption bands, reflecting GRF’s high degree of long-range molecular order. Specifically, two intense carbonyl stretching vibrations are observed at 1706 cm^−1^ and 1661 cm^−1^, which can be assigned to the lactone carbonyl within the benzofuran ring and the conjugated ketone of the cyclohexene ring, respectively. Additional characteristic features include distinct bands in the 1618–1586 cm^−1^ region associated with aromatic C=C stretching vibrations, as well as absorptions at 1213–1224 cm^−1^ corresponding to C-O stretching of the methoxy and lactone functionalities. Further bands at 1134 cm^−1^, attributed to C-C-O skeletal vibrations, and at 811 cm^−1^, arising from aromatic C-H out-of-plane bending, were also clearly detected. The presence and positions of these bands are fully consistent with previously reported ATR-FTIR spectra of GRF [30] and provide a robust spectral benchmark for identifying drug–PEDES interactions.

Before proceeding with the evaluation of possible molecular interactions within the GRF-loaded PEDES systems, it is essential to consider the complementarity of functional groups present in the molecular structures of the individual components, as intermolecular association is governed primarily by the availability of hydrogen-bond donors and acceptors. Inspection of the chemical structures shown in Figure S2(iii) (Supplementary Material) reveals that GRF contains multiple carbonyl functionalities, including lactone and ketone groups, which can act as strong hydrogen-bond acceptors, along with methoxy substituents and aromatic rings capable of participating in weaker polar and π–π interactions. Collectively, these features indicate that GRF has a high propensity to engage in diverse intermolecular interactions within a multicomponent formulation matrix.

In the case of PAA, which possesses carboxylic acid groups along its polymeric backbone, the polymer can act as both a hydrogen-bond donor and a hydrogen-bond acceptor, with a particularly strong donor capacity arising from the acidic -COOH moieties. This dual functionality enables PAA to form strong intermolecular hydrogen bonds not only with GRF carbonyl groups but also with the hydrogen-bond-rich DES components. As a consequence, PAA may preferentially interact with the DES matrix, potentially limiting the availability of interaction sites for direct drug–polymer association and thereby influencing both solubilization behavior and precipitation inhibition efficiency.

Similarly, PVP contains a lactam carbonyl group within each repeating unit, which functions as a strong hydrogen-bond acceptor but lacks hydrogen-bond donor functionality. This structural characteristic favors selective interaction with hydrogen-bond donors present in the system, including the hydroxyl and carboxylic acid groups of the DES components, as well as any available donor sites on GRF. Owing to its non-ionic and highly polar nature, PVP is therefore well positioned to engage in stabilizing interactions with GRF through complementary hydrogen bonding while remaining molecularly dispersed within the DES environment.

**Figure 8:**
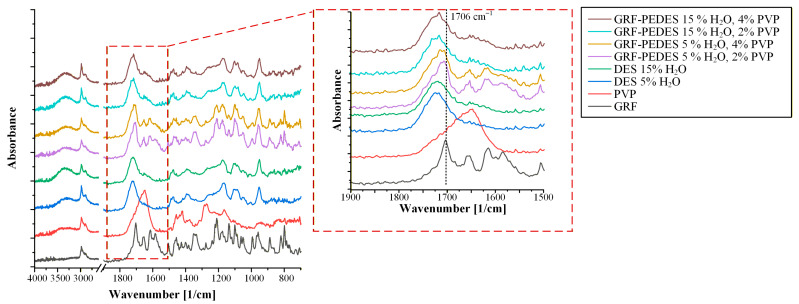
ATR-FTIR spectra of GRF-loaded PEDESs containing PVP as a PPI.

Figure 8 presents the ATR-FTIR spectra of GRF-loaded PEDES formulations containing PVP as the PPI. In the case of PEDES systems prepared with the ChCl/DL-Mal DES containing 5% *w*/*w* of water, the spectra clearly retain the principal vibrational features of GRF. Most notably, the characteristic carbonyl stretching band at 1706 cm^−1^, attributed to the lactone moiety of GRF, is distinctly observed in both formulations containing 2% and 4% *w*/*w* PVP. The absence of significant peak shifts or band broadening for GRF suggests that any potential drug-matrix interactions are insufficient to disrupt long-range molecular organization. This interpretation is consistent with the rapid recrystallization observed microscopically in these formulations. In contrast, in PEDES formulations containing 15% water, the characteristic GRF bands are no longer detectable. The disappearance of these sharp vibrational features indicates loss of the long-range crystalline organization and suggests the possible transition toward a molecularly dispersed or highly disordered drug state within the PEDES matrix. While ATR-FTIR alone cannot definitively quantify interaction strength, the spectral behavior is consistent with enhanced drug-matrix molecular interactions facilitated at higher hydration levels.

**Figure 9:**
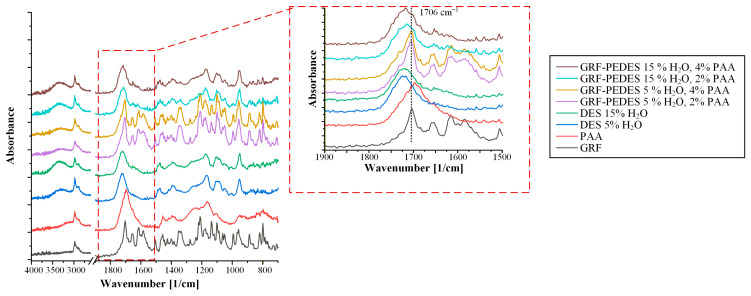
ATR-FTIR spectra of GRF-loaded PEDESs containing PAA as a PPI.

A similar trend is observed in the ATR-FTIR spectra of GRF-loaded PEDES formulations containing PAA, as shown in Figure 9.

PEDES systems prepared with 5% *w*/*w* of water again exhibit clear retention of GRF-specific vibrational features, while in PEDESs containing 15% *w*/*w* of water the characteristic GRF absorption bands are not detected. This suggests that, despite differences in polymer chemistry, sufficient hydration of the DES matrix facilitates the formation of molecular-level interactions between the API and the PEDES components. This behavior may be attributed, in part, to reduced viscosity at higher water content, which enhances molecular diffusion and facilitates intermolecular rearrangement within the PEDES matrix.

Collectively, the spectroscopic findings reinforce the hydration-threshold behavior identified during the stability studies. At low hydration (5% water), limited molecular mobility restricts effective drug-matrix association, allowing GRF to maintain crystalline order and recrystallize during storage. At higher hydration (15% water), increased structural flexibility within the eutectic network probably promotes reorganization of intermolecular interactions, facilitating molecular dispersion of GRF and suppressing nucleation. These results demonstrate that hydration governs not only solvent strength but also the structural capacity of PEDES systems to enable drug–polymer–DES interactions, while polymer functionality alone does not guarantee effective stabilization; rather, sufficient hydration is required to enable these interactions.

### 3.6. Dissolution Studies

Non-sink dissolution testing was performed to evaluate the supersaturation generation and maintenance capacity of GRF-loaded PEDES systems under dilution conditions. Unlike equilibrium solubility measurements, which describe thermodynamic limits, non-sink experiments probe the dynamic balance between supersaturation-driven nucleation and polymer-mediated precipitation inhibition. This approach is particularly appropriate for PEDES formulations, where drug release is expected to be governed by rapid solvent exchange, structural reorganization of the DES matrix, and subsequent kinetic stabilization in the aqueous phase.

To quantitatively interpret the degree of supersaturation achieved upon dilution, additional thermodynamic solubility measurements were performed in the release medium. Because dilution of DES- and PEDES-based systems introduces eutectic components into the aqueous phase, the resulting medium cannot be assumed to behave as pure PBS, pH 6.8. Even low concentrations of residual HBA/HBD components may modify drug activity coefficients through cosolvency effects, hydrogen-bond redistribution, or microenvironmental polarity shifts. Therefore, in addition to determining the equilibrium solubility of GRF in pure PBS, solubility was also measured in a diluted DES:buffer medium corresponding to a 1:10 (*v*/*v*) mixture of DES and PBS, reflecting the effective solvent composition encountered immediately after formulation dilution. This approach enables discrimination between true supersaturation relative to the relevant post-dilution thermodynamic baseline and apparent supersaturation arising from solvent-mediated solubility shifts.

**Figure 10:**
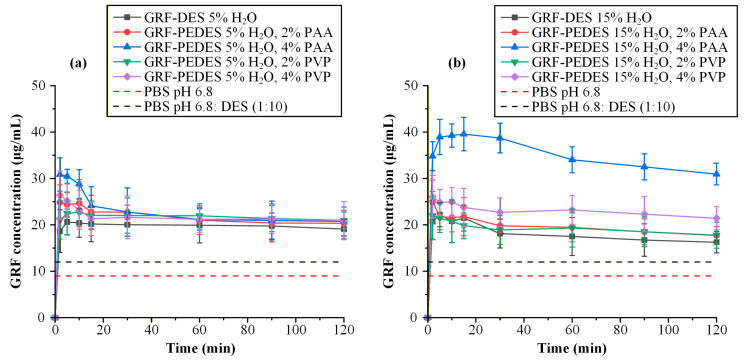
Non-sink dissolution release profiles of GRF from the pure DES formulations and the PEDESs containing 5% (**a**) or 15% *w*/*w* (**b**) water, along with the equilibrium solubilities of GRF in diluted DES:PBS pH 6.8, 1:10 *v*/*v,* and PBS pH 6.8 media.

The concentration-time profiles obtained from non-sink release experiments for both DES and PEDES formulations are presented in Figure 10, while the corresponding equilibrium GRF solubility values in PBS and in the diluted DES-containing medium are provided for direct comparison to contextualize supersaturation magnitude and decay kinetics.

Figure 10a presents the non-sink dissolution profiles of DES and PEDES formulations containing 5% *w*/*w* water. All systems generated concentrations exceeding the thermodynamic solubility of GRF in both pure PBS and the diluted DES:PBS (1:10 *v*/*v*) medium, confirming that dilution of the eutectic matrix produced a transient supersaturated state. However, none of the PEDES formulations sustained a higher supersaturation level than the polymer-free DES over extended time periods. In fact, the supersaturation decay kinetics of most PEDES systems closely paralleled the neat DES. Only the PAA-containing PEDES formulations (both 2% and 4% *w*/*w*) exhibited a modest, ‘short-lived’ elevation in drug concentration during the first ~20 min, resembling a classical “spring and parachute” profile characterized by rapid supersaturation generation followed by progressive decay.

Mechanistically, this behavior indicates that under low hydration conditions (5% *w*/*w* water), polymer incorporation does not effectively suppress nucleation and crystal growth. The initial “spring” reflects rapid solvent exchange and abrupt reduction in solvent strength upon dilution, which transiently drives the system far above its equilibrium solubility. However, the rapid concentration decline suggests that molecular mobility in the pre-dilution PEDES matrix was insufficient to promote stable drug–polymer pre-association or to generate kinetically persistent drug–polymer aggregates upon aqueous exposure. Instead, the system likely transitions rapidly toward nucleation-controlled precipitation. The slight advantage observed for PAA may arise from its dual hydrogen-bond donor/acceptor functionality, enabling transient drug–polymer association in the aqueous phase. However, at 5% hydration, the pre-organized DES network appears too rigid to facilitate cooperative restructuring during dilution. Thus, kinetic stabilization is limited and supersaturation collapses relatively quickly.

In contrast, Figure 10b demonstrates a markedly different supersaturation profile for formulations containing 15% *w*/*w* water. While the neat DES again produced a supersaturation upon dilution, the PEDES system containing 4% *w*/*w* PAA sustained significantly higher drug concentrations for the full 120 min period. This sustained elevation clearly indicates that both polymer type and polymer concentration critically modulate supersaturation performance. This observation is consistent with the established behavior of supersaturating drug delivery systems, where polymeric excipients act as precipitation inhibitors by interacting with drug molecules in solution and increasing the kinetic barrier to nucleation and crystal growth [28,44]. Notably, the effect is concentration-dependent: only at 4% PAA does the system transition into a regime of prolonged kinetic stabilization, whereas lower polymer loadings or PVP-containing systems show comparatively modest benefits.

This divergence between 5% and 15% hydration levels strongly supports the hypothesis that hydration governs the balance between thermodynamic solvent strength and kinetic stabilization capacity. At 15% water, reduced viscosity and increased configurational flexibility of the eutectic network likely facilitate pre-equilibration between GRF and polymer within the PEDES matrix prior to dilution. Consequently, upon exposure to aqueous buffer, drug–polymer associates may persist, lowering the effective molecular diffusion coefficient of free drug and increasing the kinetic barrier for critical nucleus formation. In the case of PAA at 4%, cooperative hydrogen-bonding interactions between GRF carbonyl groups and polymer carboxylic acid functionalities (along with residual DES reacting groups) may produce a dynamically reorganizing microenvironment that suppresses both nucleation rate and subsequent crystal growth.

These dissolution findings are consistent with the stability and spectroscopic data presented earlier. The 5% water systems exhibited visible GRF recrystallization during storage and retained distinct GRF vibrational features in ATR-FTIR spectra, indicating persistence of long-range molecular order and insufficient drug-matrix interaction strength. Correspondingly, their inability to maintain supersaturation upon dilution suggests that crystallization propensity is already encoded within the pre-dilution structure. Conversely, the 15% water PEDES systems showed no birefringent domains and no resolvable GRF crystalline bands in ATR-FTIR analysis, implying a molecularly dispersed or highly disordered drug state. The enhanced dissolution performance of the 4% PAA formulation therefore correlates with a preorganized, hydration-enabled interaction network capable of redistributing intermolecular forces during dilution and kinetically suppressing phase separation.

As a whole, these results demonstrate that supersaturation maintenance in PEDES systems does not arise solely from polymer presence but from a hydration-dependent cooperative regime in which sufficient molecular mobility enables effective drug–polymer–DES interaction restructuring. Polymer chemistry determines the nature of interaction (competitive vs. cooperative), polymer concentration determines interaction density, and hydration determines whether these interactions can dynamically reorganize to suppress nucleation. Thus, supersaturation performance in PEDES platforms emerges from a three-parameter interplay between water content, polymer functionality, and polymer loading, rather than from any single formulation variable alone.

### 3.7. H^1^-NMR Analysis

To clarify whether supersaturation stabilization arises from solution-phase drug–polymer association after dilution, ^1^H-NMR spectroscopy was performed in PBS (small amounts of DMSO were used to solubilize the API). Because precipitation inhibition is governed by reversible intermolecular interactions in the aqueous phase, this analysis directly probes post-dilution molecular behavior rather than pre-dilution DES structuring. Chemical shift perturbations and signal broadening were evaluated to assess potential hydrogen-bonding or hydrophobic interactions between GRF and PVP or PAA. Comparison of spectra for GRF alone and in polymer-containing media enables mechanistic correlation between dissolution performance and solution-state drug–polymer association.

**Figure 11:**
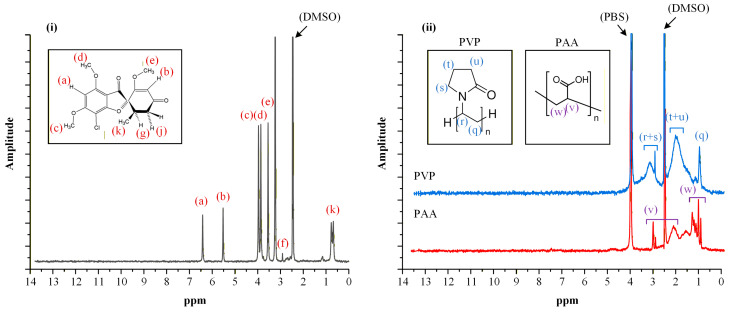
Low-frequency ^1^H-NMR spectra of GRF (**i**) and the PPIs used (**ii**).

Figure 11 shows the ^1^H-NMR spectra of neat GRF and PPIs (PVP and PAA). GRF exhibits characteristic proton NMR signals consistent with its molecular structure. An aromatic proton resonance at δ = 6.50 ppm (a) originates from the benzofuran ring system. The singlet at δ = 5.62 ppm (b) corresponds to the olefinic proton adjacent to the conjugated ketone in the cyclohexene moiety, while the assignments at δ = 4.07 (c) and 3.93 ppm (d), appearing as doublets, and a singlet at δ = 3.64 ppm (e), correspond to the methoxy substituents positioned on both the aromatic ring and lactone functionalities. A distinctive methine proton resonance at δ = 3.02 ppm (f) is consistent with a proton near the spiro junction. Diastereotopic methyl groups on the cyclohexene ring were identified through doublet resonances at δ = 0.86 and 0.79 ppm (k). These spectroscopic signatures conform with previously documented NMR data of the drug [30]. For the PPIs used, characteristic proton NMR signals were observed that were in agreement with established literature references [45,46].

**Figure 12:**
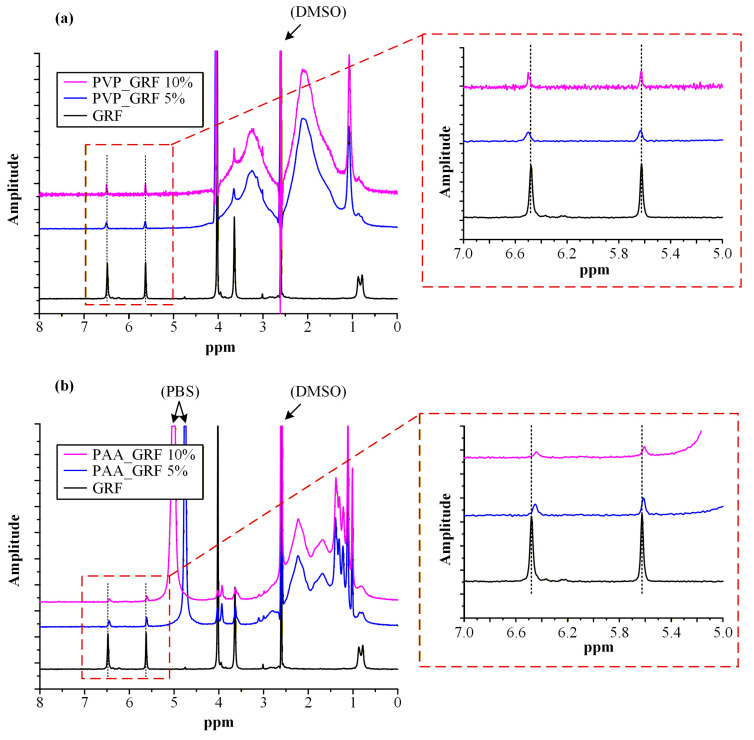
Low-frequency ^1^H-NMR spectra of GRF-PVP (**a**) and GRF–PAA (**b**) mixtures. Two different concentrations of PPIs were evaluated, namely 5 and 10% *w*/*w*.

Figure 12 shows the ^1^H-NMR spectra of neat GRF compared with those obtained in the presence of PVP and PAA (in the absence of DES components) to isolate polymer-specific effects on the electronic environment and molecular mobility of the API.

Two GRF resonances in the 5–7 ppm region exhibited systematic chemical shift perturbations upon polymer addition. These included: (i) the olefinic proton at δ = 5.62 ppm located on the cyclohexene ring between the ether and ester oxygen atoms and adjacent to the conjugated ketone, and (ii) the aromatic CH at δ = 6.50 ppm within the benzofuran benzene moiety positioned between two ether functionalities. Both protons reside in electronically sensitive environments influenced by carbonyl conjugation and heteroatom proximity, rendering them highly responsive to alterations in hydrogen-bonding interactions and local dielectric conditions.

In the presence of PVP, modest downfield shifts (to higher ppm values) were observed for these resonances. Downfield displacement indicates decreased electronic shielding, consistent with deshielding effects arising from hydrogen-bond acceptor–driven interactions between the lactam carbonyl groups of PVP and GRF-associated proton environments. Because PVP lacks hydrogen-bond donor functionality and is non-ionizable under the experimental conditions, these interactions are likely limited to dipolar and weak hydrogen-bonding associations operating in fast exchange on the NMR timescale. The relatively small magnitude of the observed shifts supports a transient and moderately weak interaction regime.

In contrast, addition of PAA induced more pronounced upfield shifts (to lower ppm values) for the same GRF protons. Upfield displacement reflects increased electronic shielding and suggests a distinct interaction mechanism. At pH 6.8, PAA exists predominantly in its ionized carboxylate form (pKa of PAA is approximately 4.2–4.5 [47], meaning that at pH 6.8 it is predominantly deprotonated, i.e., exists in the COO^−^ form), enabling strong ion-dipole and hydrogen-bond interactions with the carbonyl and ether functionalities of GRF. Such interactions can redistribute electron density within the conjugated system of GRF, increasing local shielding of adjacent olefinic and aromatic protons. Additionally, the larger magnitude of shift perturbations suggests multipoint or cooperative association, potentially accompanied by partial incorporation of GRF into polymer-associated microdomains characterized by reduced dielectric constant relative to bulk water. This microenvironmental modulation would further enhance shielding effects, particularly for the aromatic proton at δ = 6.50 ppm, which is highly sensitive to changes in ring current anisotropy and solvent polarity.

In the GRF–PAA spectra, the resonance attributable to PBS appeared at approximately 4.5–5.0 ppm. Notably, as the concentration of PAA increased within the system, this signal progressively shifted downfield. In contrast, this phenomenon was not observed in the GRF-PVP mixtures, where the PBS-associated peak remained constant at approximately 4.1 ppm and overlapped with the GRF signal. The shift in this buffer-associated signal in the presence of PAA indicates modification of proton exchange dynamics or local buffer organization. Given the polyelectrolytic nature of PAA, interaction with phosphate species and associated hydration shells may alter microenvironmental proton activity or exchange kinetics, rendering previously broadened resonances detectable. This observation further supports the conclusion that PAA does not merely interact with GRF but also reorganizes the surrounding aqueous environment, thereby influencing overall solution structure. These differences can therefore be rationalized in terms of the intrinsic physicochemical properties of the two polymers. PAA is a weak polyelectrolyte that becomes predominantly ionized under the experimental conditions (pH 6.8) [48], leading to extended chain conformations and strong interactions with surrounding solvent molecules and buffer species [49]. The presence of ionized carboxylate groups enhances its ability to participate in both hydrogen-bonding and ion–dipole interactions, thereby promoting the formation of dynamic drug–polymer–solvent interaction networks. In contrast, PVP is a neutral polymer [50,51] whose interaction capability is largely restricted to hydrogen-bond acceptance through its lactam carbonyl groups. As a result, PVP exhibits weaker interaction strength and limited ability to reorganize the surrounding aqueous microenvironment.

Taken together, the direction and magnitude of chemical shift perturbations indicate fundamentally different interaction regimes for the two polymers. PVP engages in relatively weak, primarily dipolar interactions that modestly perturb GRF electronic environments, whereas PAA forms stronger and likely multipoint associations capable of altering both drug electron density and local aqueous structuring. From a kinetic perspective, these interaction regimes may also influence the nucleation behavior of the drug upon dilution. Stronger drug–polymer association and local microenvironment restructuring in the presence of PAA are expected to decrease the activity of freely diffusing GRF molecules in solution and reduce their effective diffusion toward emerging nuclei. This effect increases the kinetic barrier for critical nucleus formation and subsequent crystal growth. In contrast, the weaker and predominantly dipolar interactions observed for PVP are less likely to significantly alter the solution microenvironment or restrict drug mobility, which may explain the comparatively lower ability of PVP-containing PEDES systems to sustain supersaturation during dissolution. These findings correlate directly with the non-sink dissolution results, where PAA (particularly at higher concentration) demonstrated superior ability to maintain supersaturation. The ^1^H-NMR data therefore provide molecular-level evidence that enhanced supersaturation stabilization in PAA-containing systems arises from stronger solution-phase drug–polymer association and microenvironmental restructuring, which collectively reduce the population of freely diffusing GRF molecules available for nucleation and subsequent crystal growth.

## 4. Conclusions

This study systematically investigated PEDES systems as liquid-state supersaturating drug delivery platforms, focusing on how controlled hydration and polymer chemistry govern both thermodynamic solubilization and kinetic stabilization. Using GRF as a crystallization-prone model compound, the work examined the influence of water content (5% and 15% *w*/*w*), polymer type (PVP versus PAA), and polymer concentration on structural organization, equilibrium solubility, storage stability, and non-sink dissolution behavior. The results demonstrate that although DESs markedly enhance drug solubility, supersaturation maintenance is not solely dictated by solvent strength. Instead, PEDES performance is governed by hydration-dependent interaction regimes. At low hydration, restricted molecular mobility limits effective drug–polymer association, resulting in recrystallization and rapid supersaturation decay. At higher hydration, increased configurational flexibility enables cooperative drug–polymer interactions (particularly in systems containing 4% PAA), leading to sustained supersaturation under non-sink conditions. Spectroscopic analyses further confirmed stronger solution-phase association between GRF and PAA relative to PVP, linking molecular-level interactions to macroscopic dissolution outcomes.

These findings establish that PEDES systems function as adaptive hydrogen-bonded liquid networks in which hydration modulates the balance between solvent structuring and dynamic interaction redistribution. The study therefore advances a rational design principle, indicating that optimal supersaturation performance requires a critical hydration threshold that permits cooperative stabilization without excessive disruption of solvent organization. However, several limitations merit consideration. Future investigations integrating rheological analysis, diffusion measurements, and in vivo performance assessment will be essential to translate these mechanistic insights into predictive formulation strategies. Nevertheless, the present work provides mechanistic insight into how hydration and polymer chemistry can be used as key formulation parameters for the rational design of DES-based supersaturating drug delivery systems.

## Figures and Tables

**Table 1:** Gravimetric analysis of PEDES formulations stored at 25 °C and 60% RH for 28 days.

Samples	Initial Weight (g)	Weight Loss (g)	Weight Loss Change (%)
PEDES 5% H_2_O, 2% PVP	22.012	−0.110	0.005
PEDES 5% H_2_O, 4% PVP	18.571	−0.130	0.007
PEDES 5% H_2_O, 2% PAA	32.400	−0.190	0.006
PEDES 5% H_2_O, 4% PAA	30.980	−0.210	0.007
PEDES 15% H_2_O, 2% PVP	31.170	−0.410	0.013
PEDES 15% H_2_O, 4% PVP	31.730	−0.430	0.014
PEDES 15% H_2_O, 2% PAA	30.900	−0.480	0.016
PEDES 15% H_2_O, 4% PAA	32.550	−0.350	0.011

**Table 2:** Saturation solubility of GRF in phosphate-buffered saline, polymer-free DESs, and PEDESs containing different water contents (5% and 15% *w*/*w*), polymer types (PVP or PAA), and polymer concentrations (2% and 4% *w*/*w*). Values are reported as mean ± SD (*n* = 3).

Samples or Medium	Saturation Solubility (μg/mL)
Buffer PBS pH 6.8	9.01 ± 1.03
DES 5% H_2_O (without polymer)	531.13 ± 11.40
DES 15% H_2_O (without polymer)	417.90 ± 6.29
PEDES 5% H_2_O, 2% PVP	486.03 ± 10.83
PEDES 5% H_2_O, 4% PVP	513.19 ± 12.72
PEDES 5% H_2_O, 2% PAA	428.61 ± 9.56
PEDES 5% H_2_O, 4% PAA	445.30 ± 6.58
PEDES 15% H_2_O, 2% PVP	363.66 ± 8.29
PEDES 15% H_2_O, 4% PVP	457.65 ± 9.31
PEDES 15% H_2_O, 2% PAA	341.77 ± 7.08
PEDES 15% H_2_O, 4% PAA	501.29 ± 5.50

**Table 3:** Gravimetric analysis of GRF-loaded PEDES formulations stored for 28 days.

Samples	Initial Weight (g)	Weight Loss (g)	Weight Loss Change (%)
GRF-PEDES 5% H_2_O, 2% PVP	25.970	−0.119	0.005
GRF-PEDES 5% H_2_O, 4% PVP	25.850	−0.160	0.006
GRF-PEDES 5% H_2_O, 2% PAA	25.510	−0.080	0.003
GRF-PEDES 5% H_2_O, 4% PAA	25.900	−0.110	0.004
GRF-PEDES 15% H_2_O, 2% PVP	25.860	−0.280	0.011
GRF-PEDES 15% H_2_O, 4% PVP	26.470	−0.320	0.012
GRF-PEDES 15% H_2_O, 2% PAA	26.130	−0.320	0.012
GRF-PEDES 15% H_2_O, 4% PAA	26.200	−0.310	0.012

## Data Availability

The original contributions presented in this study are included in the article/Supplementary Materials. Further inquiries can be directed to the corresponding author.

## Supplementary Materials

The following supporting information can be downloaded at: https://www.mdpi.com/article/10.3390/polym18060766/s1, Figure S1: PLM micrographs of the neat PEDES (i.e., without the addition of the API) during the storage stability study; Figure S2: Chemical structures of PVP (i), PAA (ii) and GRF (iii); Figure S3: ATR-FTIR spectrum of GRF.

## Abbreviations

The following abbreviations are used in this manuscript:

ANOVA	Analysis of variance
API	Active pharmaceutical ingredient
ASDs	Amorphous solid dispersions
ATR-FTIR	Attenuated total reflectance Fourier transform infrared spectroscopy
ChCl	Choline chloride
DES	Deep eutectic solvent
DL-Mal	DL-malic acid
GRF	Griseofulvin
HBA	Hydrogen bond acceptor
HBD	Hydrogen bond donor
HPLC	High-performance liquid chromatography
ILs	Ionic liquids
PEDES	Polymer-embedded deep eutectic solvent
PLM	Polarized light microscopy
PAA	Polyacrylic acid
PBS	Phosphate-buffered saline
PPIs	Polymeric precipitation inhibitors
PVP	Polyvinylpyrrolidone
PWSDs	Poorly water-soluble drugs
RH	Relative humidity
^1^H-NMR	Proton nuclear magnetic resonance
