# Supplementary material for: Polymer-Embedded Deep Eutectic Solvents: Mechanistic Insights into Storage and Supersaturation Stabilization
Source: Polymers (Basel). 2026 Mar 21;18(6):766. doi: 10.3390/polym18060766 (PMC13030360; doi:10.3390/polym18060766)
Supplement: Supplementary file 1 [file polymers-18-00766-s001.zip › polymers-4196235-supplementary.pdf]

Supplementary Materials

Polymer-Embedded Deep Eutectic Solvents: Mechanistic Insights into Storage and Supersaturation Stabilization

Afroditi Kapourani[1,*], Theodora Karyofylli-Tamisoglou[1], Ioannis Pantazos[1], Maria-Emmanouela Anagnostaki[1], Ioannis Gkougkourelas[1], Panagiotis Barmpalexis [1,2]

[1] Laboratory of Pharmaceutical Technology, Division of Pharmaceutical Technology, School of Pharmacy, Faculty of Health Sciences, Aristotle University of Thessaloniki, 541 24 Thessaloniki, Greece.

[2] Natural Products Research Centre of Excellence-AUTH (NatPro-AUTH), Center for Interdisciplinary Research and Innovation (CIRI-AUTH), Thessaloniki 57001, Greece.

Corresponding author information

*Afroditi Kapourani, PhD

Department of Pharmaceutical Technology,

School of Pharmacy, Aristotle University of Thessaloniki

Thessaloniki 54124 (Greece)

Email: akapourag@pharm.auth.gr

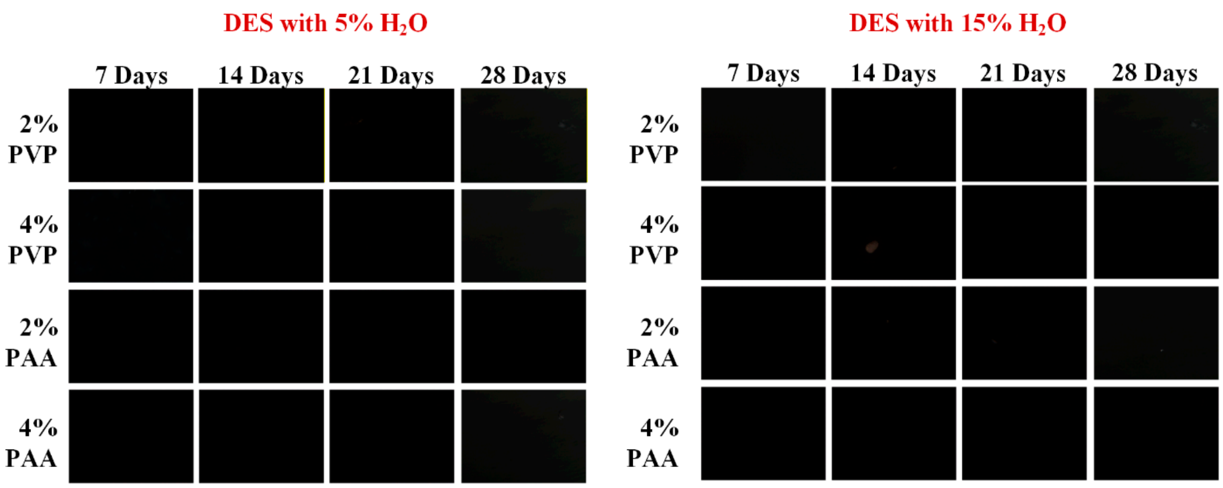

Figure S1. PLM micrographs of the neat PEDES (i.e., without the addition of the API) during the storage stability study.

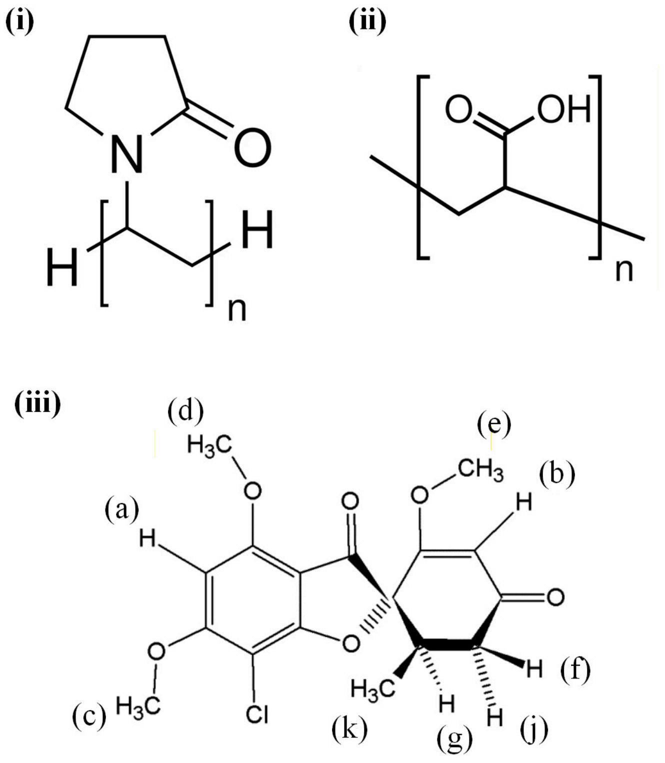

Figure S2. Chemical structures of PVP (i), PAA (ii) and GRF (iii).

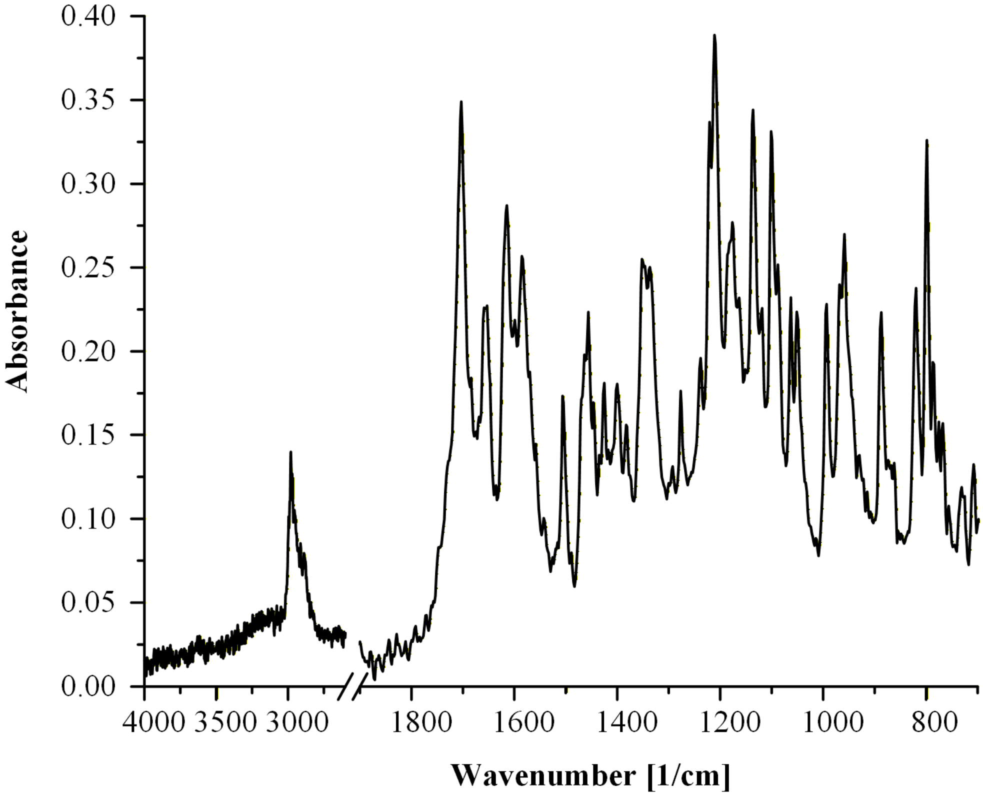

Figure S3. ATR-FTIR spectrum of GRF.
